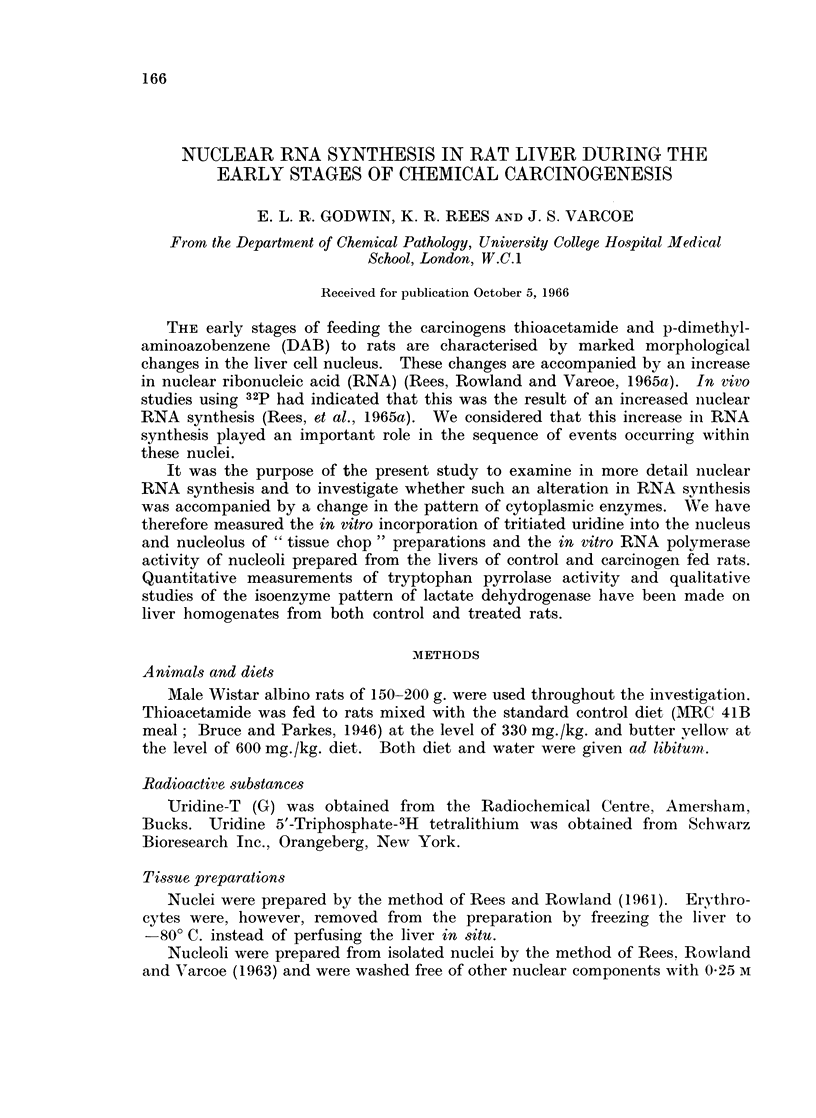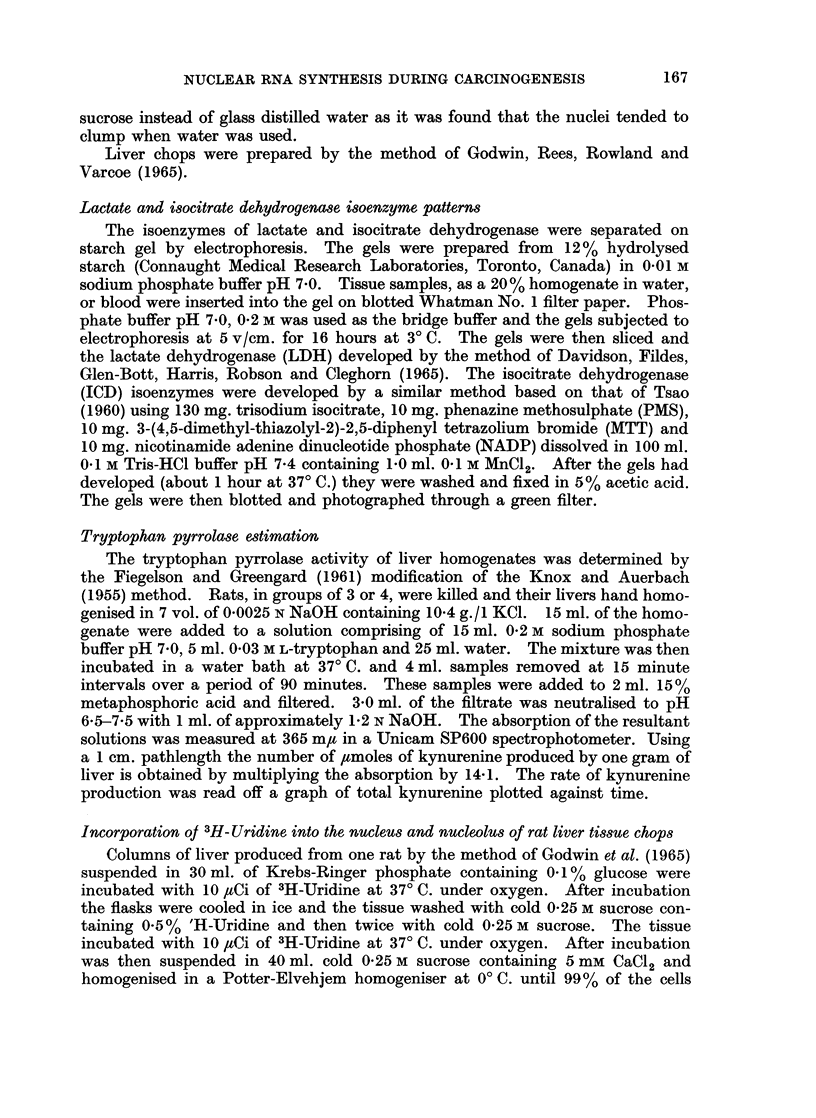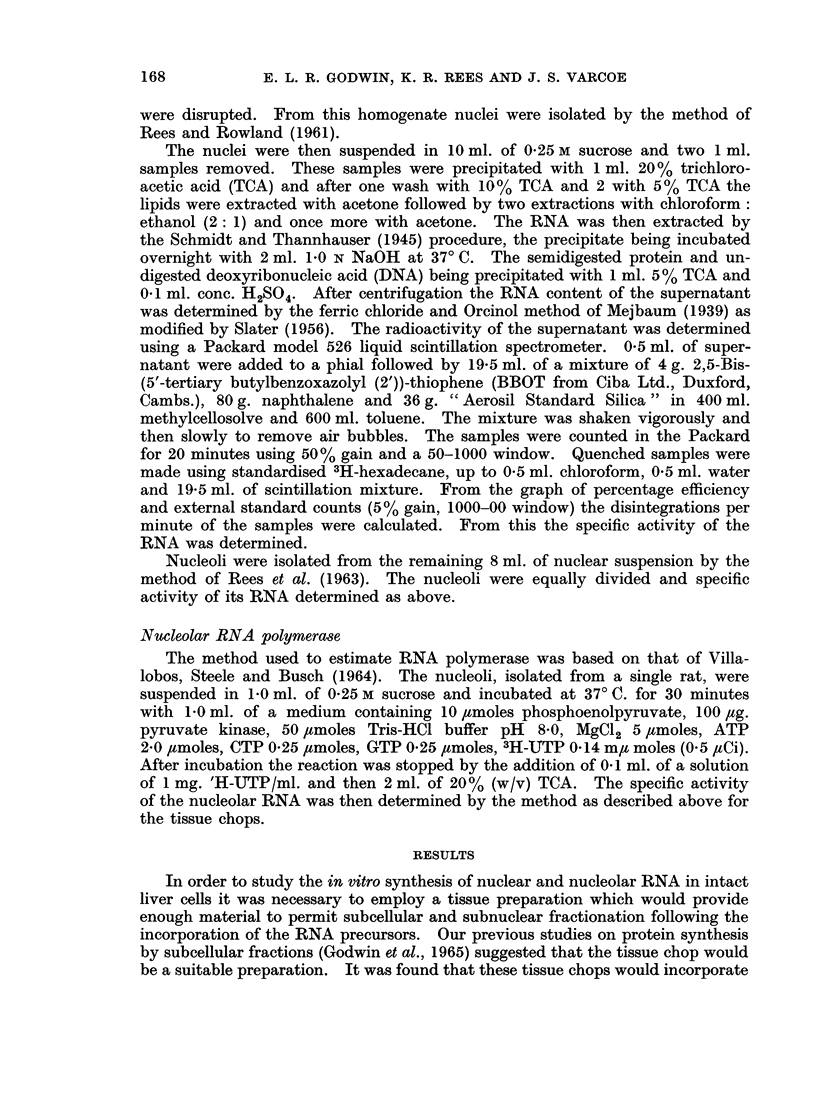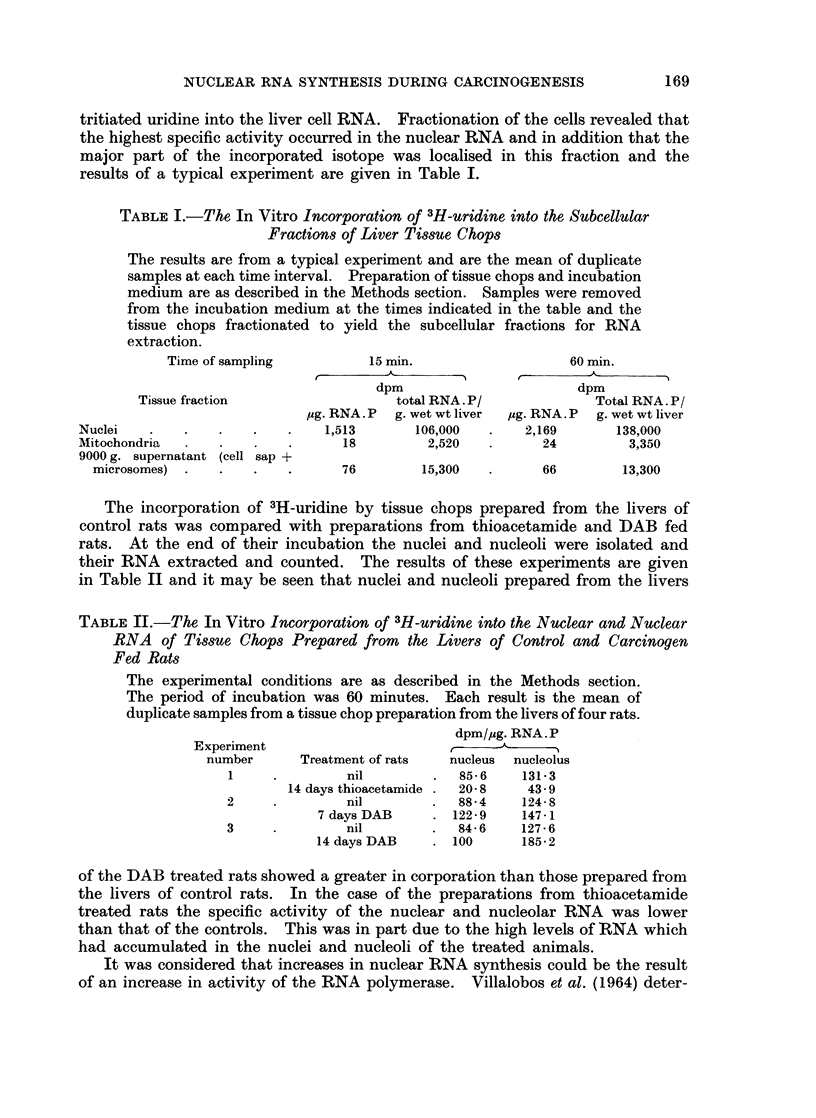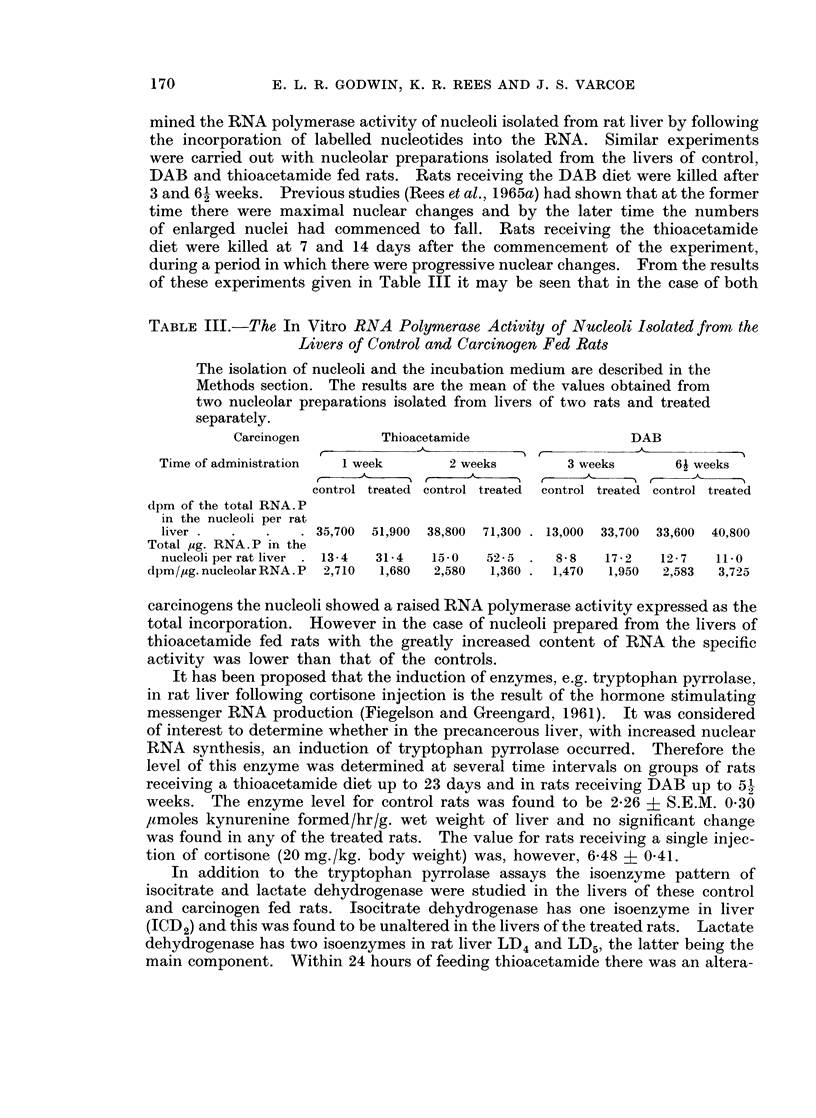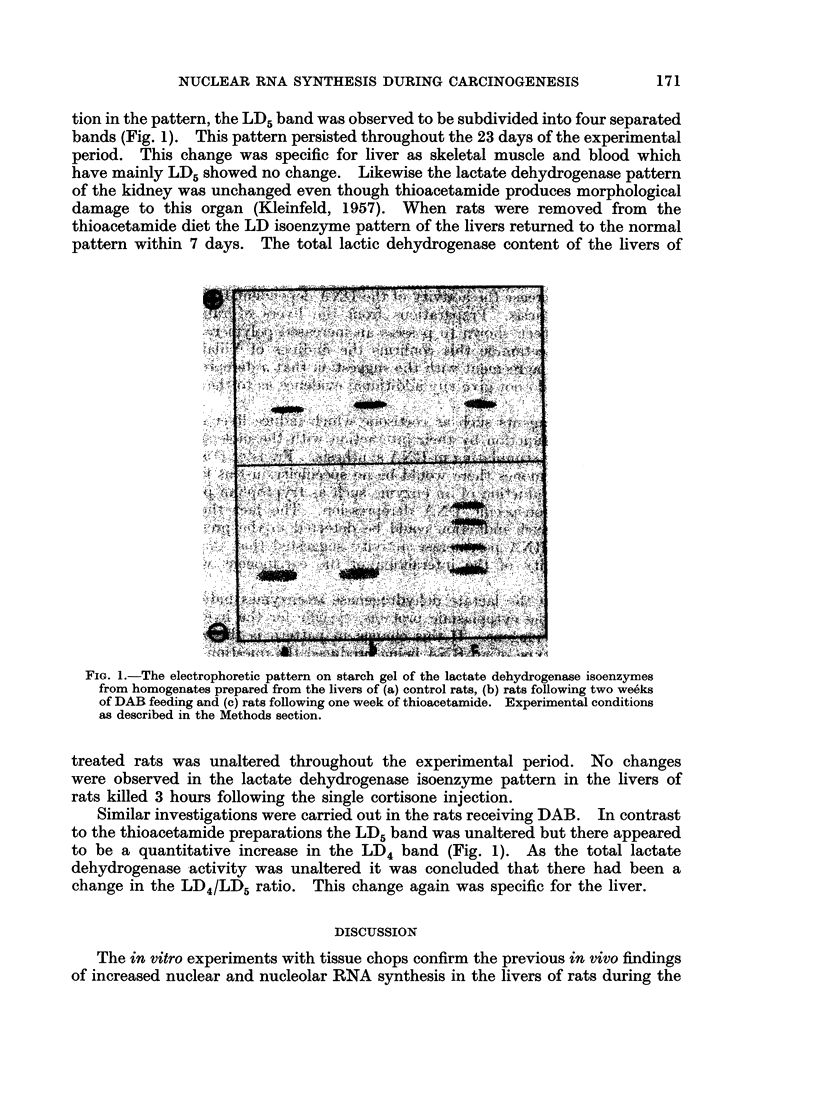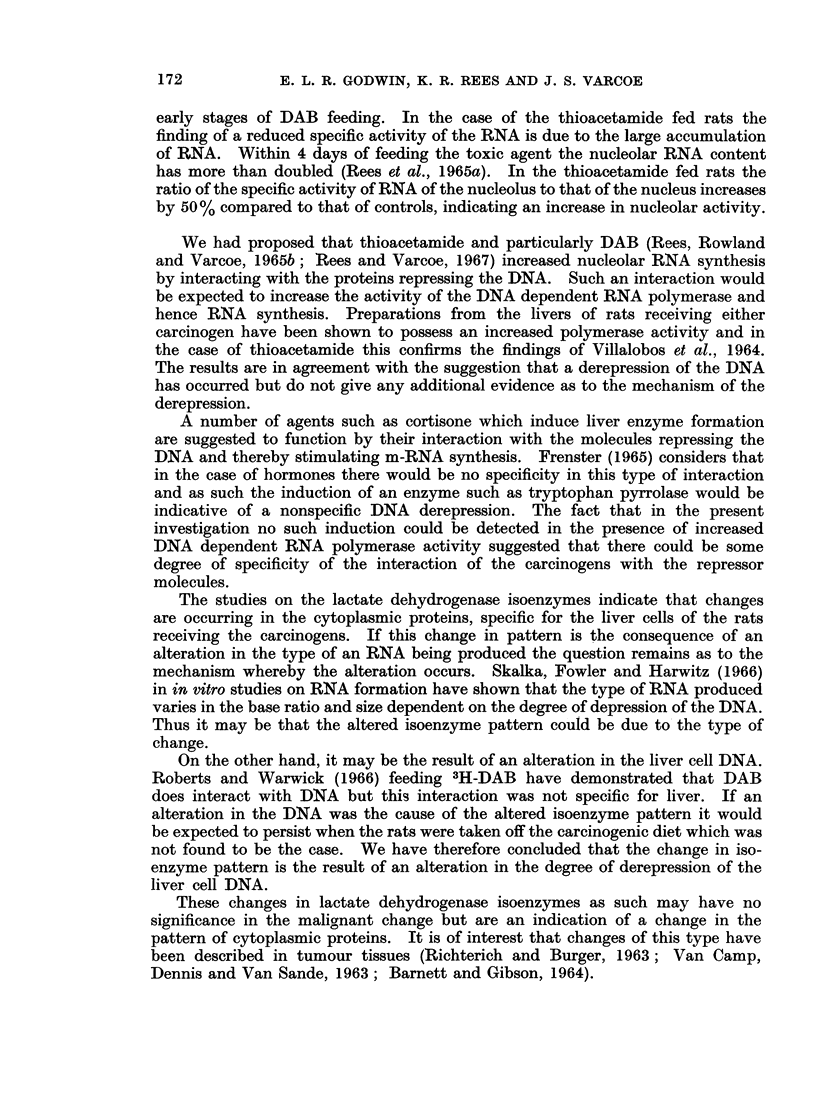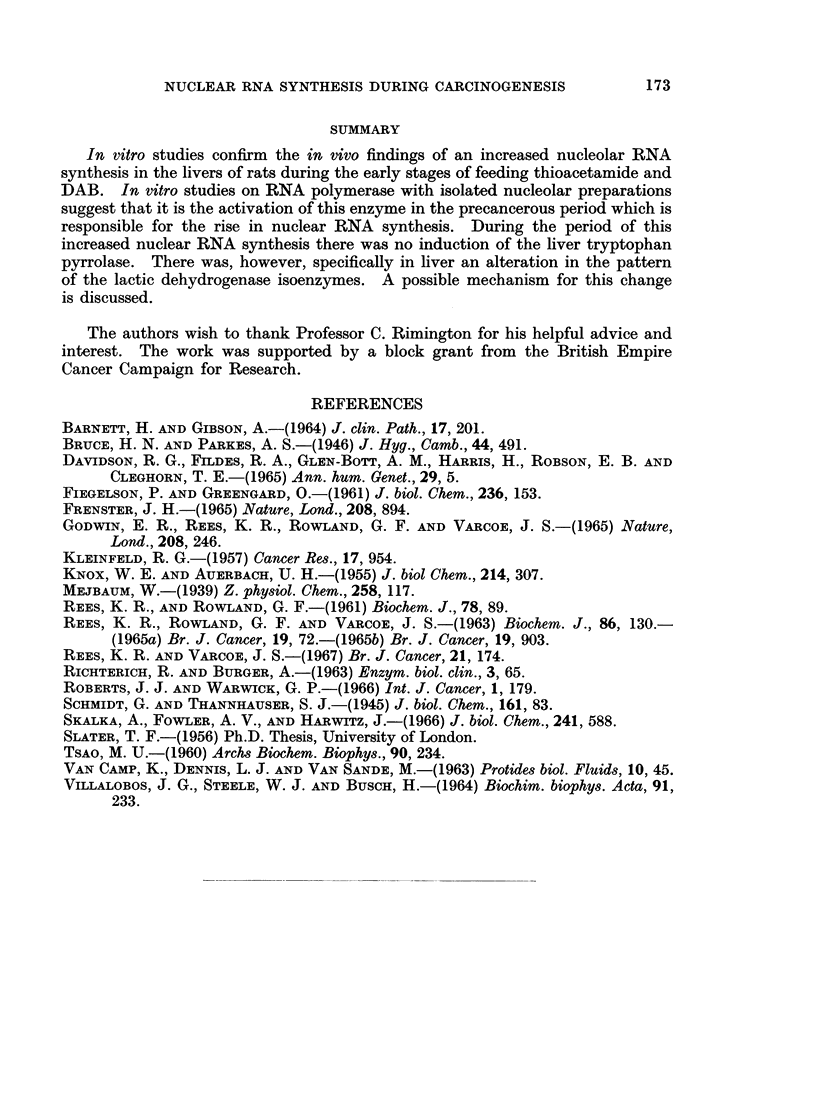# Nuclear RNA synthesis in rat liver during the early stages of chemical carcinogenesis.

**DOI:** 10.1038/bjc.1967.16

**Published:** 1967-03

**Authors:** E. L. Godwin, K. R. Rees, J. S. Varcoe


### NUCLEAR RNA SYNTHESIS IN RAT LIVER DURING THE EARLY STAGES OF CHEMICAL CARCINOGENESIS

E. L. R. GODWIN, K. R. REES AND J. S. VARCOE

*From the Department of Chemical Pathology, University College Hospital Medical School, London, W.C.1*

Received for publication October 5, 1966

THE early stages of feeding the carcinogens thioacetamide and p-dimethyl-aminoazobenzene (DAB) to rats are characterised by marked morphological changes in the liver cell nucleus. These changes are accompanied by an increase in nuclear ribonucleic acid (RNA) (Rees, Rowland and Vareoe, 1965*a*). *In vivo* studies using $^{32}P$ had indicated that this was the result of an increased nuclear RNA synthesis (Rees, *et al.*, 1965*a*). We considered that this increase in RNA synthesis played an important role in the sequence of events occurring within these nuclei.

It was the purpose of the present study to examine in more detail nuclear RNA synthesis and to investigate whether such an alteration in RNA synthesis was accompanied by a change in the pattern of cytoplasmic enzymes. We have therefore measured the *in vitro* incorporation of tritiated uridine into the nucleus and nucleolus of " tissue chop " preparations and the *in vitro* RNA polymerase activity of nucleoli prepared from the livers of control and carcinogen fed rats. Quantitative measurements of tryptophan pyrrolase activity and qualitative studies of the isoenzyme pattern of lactate dehydrogenase have been made on liver homogenates from both control and treated rats.

### METHODS

### *Animals and diets*

Male Wistar albino rats of 150–200 g. were used throughout the investigation. Thioacetamide was fed to rats mixed with the standard control diet (MRC 41B meal ; Bruce and Parkes, 1946) at the level of 330 mg./kg. and butter yellow at the level of 600 mg./kg. diet. Both diet and water were given *ad libitum*.

### *Radioactive substances*

Uridine-T (G) was obtained from the Radiochemical Centre, Amersham, Bucks. Uridine 5′-Triphosphate-$^{3}H$ tetralithium was obtained from Schwarz Bioresearch Inc., Orangeberg, New York.

### *Tissue preparations*

Nuclei were prepared by the method of Rees and Rowland (1961). Erythrocytes were, however, removed from the preparation by freezing the liver to —80° C. instead of perfusing the liver *in situ*.

Nucleoli were prepared from isolated nuclei by the method of Rees, Rowland and Varcoe (1963) and were washed free of other nuclear components with 0·25 M

sucrose instead of glass distilled water as it was found that the nuclei tended to clump when water was used.

Liver chops were prepared by the method of Godwin, Rees, Rowland and Varcoe (1965).

*Lactate and isocitrate dehydrogenase isoenzyme patterns*

The isoenzymes of lactate and isocitrate dehydrogenase were separated on starch gel by electrophoresis. The gels were prepared from 12% hydrolysed starch (Connaught Medical Research Laboratories, Toronto, Canada) in 0·01 M sodium phosphate buffer pH 7·0. Tissue samples, as a 20% homogenate in water, or blood were inserted into the gel on blotted Whatman No. 1 filter paper. Phosphate buffer pH 7·0, 0·2 M was used as the bridge buffer and the gels subjected to electrophoresis at 5 v/cm. for 16 hours at 3° C. The gels were then sliced and the lactate dehydrogenase (LDH) developed by the method of Davidson, Fildes, Glen-Bott, Harris, Robson and Cleghorn (1965). The isocitrate dehydrogenase (ICD) isoenzymes were developed by a similar method based on that of Tsao (1960) using 130 mg. trisodium isocitrate, 10 mg. phenazine methosulphate (PMS), 10 mg. 3-(4,5-dimethyl-thiazolyl-2)-2,5-diphenyl tetrazolium bromide (MTT) and 10 mg. nicotinamide adenine dinucleotide phosphate (NADP) dissolved in 100 ml. 0·1 M Tris-HCl buffer pH 7·4 containing 1·0 ml. 0·1 M $MnCl_2$. After the gels had developed (about 1 hour at 37° C.) they were washed and fixed in 5% acetic acid. The gels were then blotted and photographed through a green filter.

*Tryptophan pyrrolase estimation*

The tryptophan pyrrolase activity of liver homogenates was determined by the Fiegelson and Greengard (1961) modification of the Knox and Auerbach (1955) method. Rats, in groups of 3 or 4, were killed and their livers hand homogenised in 7 vol. of 0·0025 N NaOH containing 10·4 g./l KCl. 15 ml. of the homogenate were added to a solution comprising of 15 ml. 0·2 M sodium phosphate buffer pH 7·0, 5 ml. 0·03 M L-tryptophan and 25 ml. water. The mixture was then incubated in a water bath at 37° C. and 4 ml. samples removed at 15 minute intervals over a period of 90 minutes. These samples were added to 2 ml. 15% metaphosphoric acid and filtered. 3·0 ml. of the filtrate was neutralised to pH 6·5–7·5 with 1 ml. of approximately 1·2 N NaOH. The absorption of the resultant solutions was measured at 365 m$\mu$ in a Unicam SP600 spectrophotometer. Using a 1 cm. pathlength the number of $\mu$moles of kynurenine produced by one gram of liver is obtained by multiplying the absorption by 14·1. The rate of kynurenine production was read off a graph of total kynurenine plotted against time.

*Incorporation of $^3$H-Uridine into the nucleus and nucleolus of rat liver tissue chops*

Columns of liver produced from one rat by the method of Godwin *et al.* (1965) suspended in 30 ml. of Krebs-Ringer phosphate containing 0·1% glucose were incubated with 10 $\mu$Ci of $^3$H-Uridine at 37° C. under oxygen. After incubation the flasks were cooled in ice and the tissue washed with cold 0·25 M sucrose containing 0·5% 'H-Uridine and then twice with cold 0·25 M sucrose. The tissue incubated with 10 $\mu$Ci of $^3$H-Uridine at 37° C. under oxygen. After incubation was then suspended in 40 ml. cold 0·25 M sucrose containing 5 mM $CaCl_2$ and homogenised in a Potter-Elvehjem homogeniser at 0° C. until 99% of the cells were disrupted. From this homogenate nuclei were isolated by the method of Rees and Rowland (1961).

The nuclei were then suspended in 10 ml. of 0·25 M sucrose and two 1 ml. samples removed. These samples were precipitated with 1 ml. 20% trichloroacetic acid (TCA) and after one wash with 10% TCA and 2 with 5% TCA the lipids were extracted with acetone followed by two extractions with chloroform : ethanol (2 : 1) and once more with acetone. The RNA was then extracted by the Schmidt and Thannhauser (1945) procedure, the precipitate being incubated overnight with 2 ml. 1·0 N NaOH at 37° C. The semidigested protein and undigested deoxyribonucleic acid (DNA) being precipitated with 1 ml. 5% TCA and 0·1 ml. conc. $H_2SO_4$. After centrifugation the RNA content of the supernatant was determined by the ferric chloride and Orcinol method of Mejbaum (1939) as modified by Slater (1956). The radioactivity of the supernatant was determined using a Packard model 526 liquid scintillation spectrometer. 0·5 ml. of supernatant were added to a phial followed by 19·5 ml. of a mixture of 4 g. 2,5-Bis-(5′-tertiary butylbenzoxazolyl (2′))-thiophene (BBOT from Ciba Ltd., Duxford, Cambs.), 80 g. naphthalene and 36 g. "Aerosil Standard Silica" in 400 ml. methylcellosolve and 600 ml. toluene. The mixture was shaken vigorously and then slowly to remove air bubbles. The samples were counted in the Packard for 20 minutes using 50% gain and a 50–1000 window. Quenched samples were made using standardised $^3$H-hexadecane, up to 0·5 ml. chloroform, 0·5 ml. water and 19·5 ml. of scintillation mixture. From the graph of percentage efficiency and external standard counts (5% gain, 1000–00 window) the disintegrations per minute of the samples were calculated. From this the specific activity of the RNA was determined.

Nucleoli were isolated from the remaining 8 ml. of nuclear suspension by the method of Rees *et al.* (1963). The nucleoli were equally divided and specific activity of its RNA determined as above.

*Nucleolar RNA polymerase*

The method used to estimate RNA polymerase was based on that of Villalobos, Steele and Busch (1964). The nucleoli, isolated from a single rat, were suspended in 1·0 ml. of 0·25 M sucrose and incubated at 37° C. for 30 minutes with 1·0 ml. of a medium containing 10 $\mu$moles phosphoenolpyruvate, 100 $\mu$g. pyruvate kinase, 50 $\mu$moles Tris-HCl buffer pH 8·0, $MgCl_2$ 5 $\mu$moles, ATP 2·0 $\mu$moles, CTP 0·25 $\mu$moles, GTP 0·25 $\mu$moles, $^3$H-UTP 0·14 m$\mu$ moles (0·5 $\mu$Ci). After incubation the reaction was stopped by the addition of 0·1 ml. of a solution of 1 mg. ′H-UTP/ml. and then 2 ml. of 20% (w/v) TCA. The specific activity of the nucleolar RNA was then determined by the method as described above for the tissue chops.

### RESULTS

In order to study the *in vitro* synthesis of nuclear and nucleolar RNA in intact liver cells it was necessary to employ a tissue preparation which would provide enough material to permit subcellular and subnuclear fractionation following the incorporation of the RNA precursors. Our previous studies on protein synthesis by subcellular fractions (Godwin *et al.*, 1965) suggested that the tissue chop would be a suitable preparation. It was found that these tissue chops would incorporate tritiated uridine into the liver cell RNA. Fractionation of the cells revealed that the highest specific activity occurred in the nuclear RNA and in addition that the major part of the incorporated isotope was localised in this fraction and the results of a typical experiment are given in Table I.

TABLE I.—*The* In Vitro *Incorporation of* $^3$*H-uridine into the Subcellular Fractions of Liver Tissue Chops*

The results are from a typical experiment and are the mean of duplicate samples at each time interval. Preparation of tissue chops and incubation medium are as described in the Methods section. Samples were removed from the incubation medium at the times indicated in the table and the tissue chops fractionated to yield the subcellular fractions for RNA extraction.

| Time of sampling | 15 min. | | 60 min. | |
|---|---|---|---|---|
| | dpm | | dpm | |
| Tissue fraction | μg. RNA.P | total RNA.P/ g. wet wt liver | μg. RNA.P | Total RNA.P/ g. wet wt liver |
| Nuclei | 1,513 | 106,000 | 2,169 | 138,000 |
| Mitochondria | 18 | 2,520 | 24 | 3,350 |
| 9000 g. supernatant (cell sap + microsomes) | 76 | 15,300 | 66 | 13,300 |

The incorporation of $^3$H-uridine by tissue chops prepared from the livers of control rats was compared with preparations from thioacetamide and DAB fed rats. At the end of their incubation the nuclei and nucleoli were isolated and their RNA extracted and counted. The results of these experiments are given in Table II and it may be seen that nuclei and nucleoli prepared from the livers of the DAB treated rats showed a greater in corporation than those prepared from the livers of control rats. In the case of the preparations from thioacetamide treated rats the specific activity of the nuclear and nucleolar RNA was lower than that of the controls. This was in part due to the high levels of RNA which had accumulated in the nuclei and nucleoli of the treated animals.

TABLE II.—*The* In Vitro *Incorporation of* $^3$*H-uridine into the Nuclear and Nuclear RNA of Tissue Chops Prepared from the Livers of Control and Carcinogen Fed Rats*

The experimental conditions are as described in the Methods section. The period of incubation was 60 minutes. Each result is the mean of duplicate samples from a tissue chop preparation from the livers of four rats.

| Experiment number | Treatment of rats | dpm/μg. RNA.P nucleus | nucleolus |
|---|---|---|---|
| 1 | nil | 85·6 | 131·3 |
| | 14 days thioacetamide | 20·8 | 43·9 |
| 2 | nil | 88·4 | 124·8 |
| | 7 days DAB | 122·9 | 147·1 |
| 3 | nil | 84·6 | 127·6 |
| | 14 days DAB | 100 | 185·2 |

It was considered that increases in nuclear RNA synthesis could be the result of an increase in activity of the RNA polymerase. Villalobos *et al.* (1964) determined the RNA polymerase activity of nucleoli isolated from rat liver by following the incorporation of labelled nucleotides into the RNA. Similar experiments were carried out with nucleolar preparations isolated from the livers of control, DAB and thioacetamide fed rats. Rats receiving the DAB diet were killed after 3 and 6½ weeks. Previous studies (Rees *et al.*, 1965*a*) had shown that at the former time there were maximal nuclear changes and by the later time the numbers of enlarged nuclei had commenced to fall. Rats receiving the thioacetamide diet were killed at 7 and 14 days after the commencement of the experiment, during a period in which there were progressive nuclear changes. From the results of these experiments given in Table III it may be seen that in the case of both

TABLE III.—*The* In Vitro *RNA Polymerase Activity of Nucleoli Isolated from the Livers of Control and Carcinogen Fed Rats*

The isolation of nucleoli and the incubation medium are described in the Methods section. The results are the mean of the values obtained from two nucleolar preparations isolated from livers of two rats and treated separately.

| Carcinogen | Thioacetamide | | | | DAB | | | |
|---|---|---|---|---|---|---|---|---|
| Time of administration | 1 week | | 2 weeks | | 3 weeks | | 6½ weeks | |
| | control | treated | control | treated | control | treated | control | treated |
| dpm of the total RNA.P in the nucleoli per rat liver . . . . | 35,700 | 51,900 | 38,800 | 71,300 . | 13,000 | 33,700 | 33,600 | 40,800 |
| Total $\mu$g. RNA.P in the nucleoli per rat liver . | 13·4 | 31·4 | 15·0 | 52·5 . | 8·8 | 17·2 | 12·7 | 11·0 |
| dpm/$\mu$g. nucleolar RNA.P | 2,710 | 1,680 | 2,580 | 1,360 . | 1,470 | 1,950 | 2,583 | 3,725 |

carcinogens the nucleoli showed a raised RNA polymerase activity expressed as the total incorporation. However in the case of nucleoli prepared from the livers of thioacetamide fed rats with the greatly increased content of RNA the specific activity was lower than that of the controls.

It has been proposed that the induction of enzymes, e.g. tryptophan pyrrolase, in rat liver following cortisone injection is the result of the hormone stimulating messenger RNA production (Fiegelson and Greengard, 1961). It was considered of interest to determine whether in the precancerous liver, with increased nuclear RNA synthesis, an induction of tryptophan pyrrolase occurred. Therefore the level of this enzyme was determined at several time intervals on groups of rats receiving a thioacetamide diet up to 23 days and in rats receiving DAB up to 5½ weeks. The enzyme level for control rats was found to be 2·26 $\pm$ S.E.M. 0·30 $\mu$moles kynurenine formed/hr/g. wet weight of liver and no significant change was found in any of the treated rats. The value for rats receiving a single injection of cortisone (20 mg./kg. body weight) was, however, 6·48 $\pm$ 0·41.

In addition to the tryptophan pyrrolase assays the isoenzyme pattern of isocitrate and lactate dehydrogenase were studied in the livers of these control and carcinogen fed rats. Isocitrate dehydrogenase has one isoenzyme in liver ($ICD_2$) and this was found to be unaltered in the livers of the treated rats. Lactate dehydrogenase has two isoenzymes in rat liver $LD_4$ and $LD_5$, the latter being the main component. Within 24 hours of feeding thioacetamide there was an alteration in the pattern, the $LD_5$ band was observed to be subdivided into four separated bands (Fig. 1). This pattern persisted throughout the 23 days of the experimental period. This change was specific for liver as skeletal muscle and blood which have mainly $LD_5$ showed no change. Likewise the lactate dehydrogenase pattern of the kidney was unchanged even though thioacetamide produces morphological damage to this organ (Kleinfeld, 1957). When rats were removed from the thioacetamide diet the LD isoenzyme pattern of the livers returned to the normal pattern within 7 days. The total lactic dehydrogenase content of the livers of

FIG. 1.—The electrophoretic pattern on starch gel of the lactate dehydrogenase isoenzymes from homogenates prepared from the livers of (a) control rats, (b) rats following two weeks of DAB feeding and (c) rats following one week of thioacetamide. Experimental conditions as described in the Methods section.

treated rats was unaltered throughout the experimental period. No changes were observed in the lactate dehydrogenase isoenzyme pattern in the livers of rats killed 3 hours following the single cortisone injection.

Similar investigations were carried out in the rats receiving DAB. In contrast to the thioacetamide preparations the $LD_5$ band was unaltered but there appeared to be a quantitative increase in the $LD_4$ band (Fig. 1). As the total lactate dehydrogenase activity was unaltered it was concluded that there had been a change in the $LD_4/LD_5$ ratio. This change again was specific for the liver.

### DISCUSSION

The *in vitro* experiments with tissue chops confirm the previous *in vivo* findings of increased nuclear and nucleolar RNA synthesis in the livers of rats during the early stages of DAB feeding. In the case of the thioacetamide fed rats the finding of a reduced specific activity of the RNA is due to the large accumulation of RNA. Within 4 days of feeding the toxic agent the nucleolar RNA content has more than doubled (Rees *et al.*, 1965*a*). In the thioacetamide fed rats the ratio of the specific activity of RNA of the nucleolus to that of the nucleus increases by 50% compared to that of controls, indicating an increase in nucleolar activity.

We had proposed that thioacetamide and particularly DAB (Rees, Rowland and Varcoe, 1965*b*; Rees and Varcoe, 1967) increased nucleolar RNA synthesis by interacting with the proteins repressing the DNA. Such an interaction would be expected to increase the activity of the DNA dependent RNA polymerase and hence RNA synthesis. Preparations from the livers of rats receiving either carcinogen have been shown to possess an increased polymerase activity and in the case of thioacetamide this confirms the findings of Villalobos *et al.*, 1964. The results are in agreement with the suggestion that a derepression of the DNA has occurred but do not give any additional evidence as to the mechanism of the derepression.

A number of agents such as cortisone which induce liver enzyme formation are suggested to function by their interaction with the molecules repressing the DNA and thereby stimulating m-RNA synthesis. Frenster (1965) considers that in the case of hormones there would be no specificity in this type of interaction and as such the induction of an enzyme such as tryptophan pyrrolase would be indicative of a nonspecific DNA derepression. The fact that in the present investigation no such induction could be detected in the presence of increased DNA dependent RNA polymerase activity suggested that there could be some degree of specificity of the interaction of the carcinogens with the repressor molecules.

The studies on the lactate dehydrogenase isoenzymes indicate that changes are occurring in the cytoplasmic proteins, specific for the liver cells of the rats receiving the carcinogens. If this change in pattern is the consequence of an alteration in the type of an RNA being produced the question remains as to the mechanism whereby the alteration occurs. Skalka, Fowler and Harwitz (1966) in *in vitro* studies on RNA formation have shown that the type of RNA produced varies in the base ratio and size dependent on the degree of depression of the DNA. Thus it may be that the altered isoenzyme pattern could be due to the type of change.

On the other hand, it may be the result of an alteration in the liver cell DNA. Roberts and Warwick (1966) feeding $^{3}$H-DAB have demonstrated that DAB does interact with DNA but this interaction was not specific for liver. If an alteration in the DNA was the cause of the altered isoenzyme pattern it would be expected to persist when the rats were taken off the carcinogenic diet which was not found to be the case. We have therefore concluded that the change in isoenzyme pattern is the result of an alteration in the degree of derepression of the liver cell DNA.

These changes in lactate dehydrogenase isoenzymes as such may have no significance in the malignant change but are an indication of a change in the pattern of cytoplasmic proteins. It is of interest that changes of this type have been described in tumour tissues (Richterich and Burger, 1963; Van Camp, Dennis and Van Sande, 1963; Barnett and Gibson, 1964).

SUMMARY

*In vitro* studies confirm the *in vivo* findings of an increased nucleolar RNA synthesis in the livers of rats during the early stages of feeding thioacetamide and DAB. *In vitro* studies on RNA polymerase with isolated nucleolar preparations suggest that it is the activation of this enzyme in the precancerous period which is responsible for the rise in nuclear RNA synthesis. During the period of this increased nuclear RNA synthesis there was no induction of the liver tryptophan pyrrolase. There was, however, specifically in liver an alteration in the pattern of the lactic dehydrogenase isoenzymes. A possible mechanism for this change is discussed.

The authors wish to thank Professor C. Rimington for his helpful advice and interest. The work was supported by a block grant from the British Empire Cancer Campaign for Research.

REFERENCES

BARNETT, H. AND GIBSON, A.—(1964) *J. clin. Path.*, **17**, 201.
BRUCE, H. N. AND PARKES, A. S.—(1946) *J. Hyg., Camb.*, **44**, 491.
DAVIDSON, R. G., FILDES, R. A., GLEN-BOTT, A. M., HARRIS, H., ROBSON, E. B. AND CLEGHORN, T. E.—(1965) *Ann. hum. Genet.*, **29**, 5.
FIEGELSON, P. AND GREENGARD, O.—(1961) *J. biol. Chem.*, **236**, 153.
FRENSTER, J. H.—(1965) *Nature, Lond.*, **208**, 894.
GODWIN, E. R., REES, K. R., ROWLAND, G. F. AND VARCOE, J. S.—(1965) *Nature, Lond.*, **208**, 246.
KLEINFELD, R. G.—(1957) *Cancer Res.*, **17**, 954.
KNOX, W. E. AND AUERBACH, U. H.—(1955) *J. biol Chem.*, **214**, 307.
MEJBAUM, W.—(1939) *Z. physiol. Chem.*, **258**, 117.
REES, K. R., AND ROWLAND, G. F.—(1961) *Biochem. J.*, **78**, 89.
REES, K. R., ROWLAND, G. F. AND VARCOE, J. S.—(1963) *Biochem. J.*, **86**, 130.—(1965*a*) *Br. J. Cancer*, **19**, 72.—(1965*b*) *Br. J. Cancer*, **19**, 903.
REES, K. R. AND VARCOE, J. S.—(1967) *Br. J. Cancer*, **21**, 174.
RICHTERICH, R. AND BURGER, A.—(1963) *Enzym. biol. clin.*, **3**, 65.
ROBERTS, J. J. AND WARWICK, G. P.—(1966) *Int. J. Cancer*, **1**, 179.
SCHMIDT, G. AND THANNHAUSER, S. J.—(1945) *J. biol. Chem.*, **161**, 83.
SKALKA, A., FOWLER, A. V., AND HARWITZ, J.—(1966) *J. biol. Chem.*, **241**, 588.
SLATER, T. F.—(1956) Ph.D. Thesis, University of London.
TSAO, M. U.—(1960) *Archs Biochem. Biophys.*, **90**, 234.
VAN CAMP, K., DENNIS, L. J. AND VAN SANDE, M.—(1963) *Protides biol. Fluids*, **10**, 45.
VILLALOBOS, J. G., STEELE, W. J. AND BUSCH, H.—(1964) *Biochim. biophys. Acta*, **91**, 233.